# Solitary intrathyroidal metastasis of renal clear cell carcinoma in a toxic substernal multinodular goiter

**DOI:** 10.1186/1756-6614-1-6

**Published:** 2008-10-24

**Authors:** Gianlorenzo Dionigi, Silvia Uccella, Myriam Gandolfo, Adriana Lai, Valentina Bertocchi, Francesca Rovera, Maria Laura Tanda

**Affiliations:** 1 Department of Surgical Sciences, University of Insubria, Varese, Italy; 2 Human Morphology, University of Insubria, Varese, Italy; 3 Clinical Medicine, University of Insubria, Varese, Italy

## Abstract

**Introduction:**

Thyroid gland is a rare site of clinically detectable tumor metastasis.

**Case report:**

A 71-year-old woman was referred to our department for an evaluation of toxic multinodular substernal goiter. She had a history of renal clear cell carcinoma of the left kidney, which had been resected 2 years previously. US confirmed the multinodular goiter. Total thyroidectomy with neuromonitoring was performed on March 2008. A histological examination revealed a solitary metastasis of a clear cell renal cancer in a diffuse multinodular goiter. No distant metastases are detected.

**Conclusion:**

Although uncommon, it is important for the endocrine surgeon and endocrine oncologist to be able to recognize and differentiate intrathyroid metastases from more primary common thyroid neoplasms. The diagnosis can be suspected if the patient has a thyroid tumor and a past history of extrathyroid cancer. These tumors, on the whole, tend to behave more aggressively and, in most cases, the use of multimodality therapy is recommended.

## Introduction

Thyroid cancer refers to any kind of malignant tumors of the thyroid gland [1,2]. Thyroid cancers can be classified according to their pathological characteristics and origin in primary and secondary (i.e. metastases) [1,2].

The four main types of primary thyroid cancer are papillary, follicular, medullary, and anaplastic thyroid cancer [3]. Papillary and follicular carcinoma are the most common form of differentiated follicular cell-derived carcinomas and comprises 90–95% of all newly diagnosed thyroid cancers [3]. Medullary thyroid cancer is a rare and aggressive type of cancer deriving from the parafollicular cells and accounting for 5% of all thyroid carcinomas [4]. Other rare types of primary thyroid malignant tumors are squamous cell carcinoma, mucoepidermoid carcinoma, sclerosing mucoepidermoid with eosinophilia, teratomas, mucinous carcinoma, spindle cell tumor, lymphomas and carcinoma showing thymus-like element [5-13]. The thyroid gland is a rare site of clinically detectable tumor metastasis: a palpable thyroid tumor is usually assumed to be a primary thyroid tumor [14].

This report describes herein a patient with solitary metastasis to the thyroid, who had undergone a left nephrectomy for renal clear cell carcinoma 2 years previously. Unique pathological figures are presented.

## Case report

A 71-year-old woman underwent nephrectomy of the right kidney for renal clear cell carcinoma on September 26, 2006. The tumor measured 28 mm in size and was localized in the upper pole of the right kidney. The tumor had been incidentally demonstrated on routine ultrasonography (US). Preoperative whole body CT-scan was negative for local and distant metastases. In the final histological examination, the tumor extended into a renal vein, regional nodes could not be assessed (pT3b, pNX, G2). The surgical margin was free of the tumor. Her postoperative course was uneventful. Neither postoperative adjuvant chemotherapy nor interferon was given.

In September 2006, she was also referred to the Department of Clinical Medicine, Division of Endocrinology, for an evaluation of toxic substernal goiter with chronic thyroiditis. She had no complaints that could be related to thyroid dysfunction, nor stridor, hoarseness, dysphagia. A multinodular goiter was noticed at palpation. The patient underwent antithyroid medication with methimazole.

On January 2008 the patient underwent new endocrine consultation. Her general health condition was excellent. She was euthyroid and all laboratory data were normal. US demonstrated diffuse, bilateral and well-demarcated micro and micronodules both normooechoic and hypoechoic containing high-echo spots representing small calcifications measuring 13 × 14 × 18 mm, 14 × 17 × 17 mm, 16 × 16 × 14 mm and 16 × 22 × 20 mm. She underwent a total thyroidectomy with intraoperative neuromonitoring (NIM-Response 2.0 System, Medtronic Xomed, Jacksonville, Florida).

Five mm-thick sections were stained with hematoxylin-eosin (H&E) for histopathologic examination. Additional 3 mm-thick sections, collected on positively charges slides (SuperFrost^®^Plus, Menzel GmbH & Co KG, Braunschweig) were used for immunohistochemical analysis. The immunostainings for CD10 (clone 56 CG) and TTF-1 (Thyroid Transcription Factor, clone 8G7G3/1) were performed with an automated immunostainer (Benchmark XT, Ventana Medical Systems).

At macroscopic examination the thyroid was enlarged, with a distorted shape, the left lobe being larger than the right one. On cross section, the gland was occupied by multiple nodules, some of which were partially cystic and showed areas of calcification and haemorrhage. A histological diagnosis of nodular hyperplasia was formulated.

**Figure 1:**
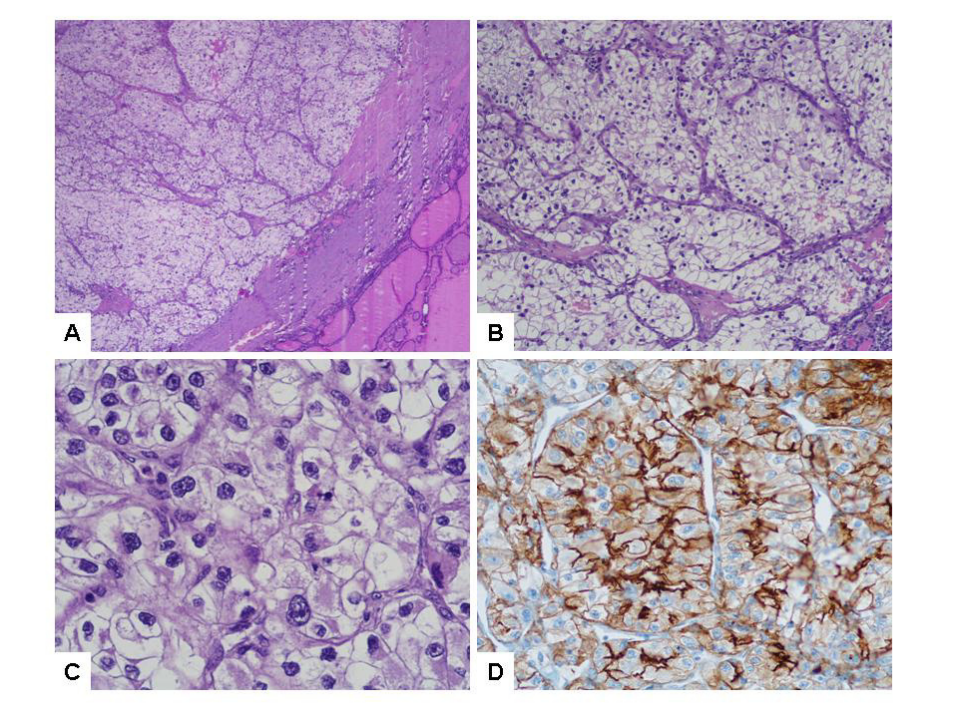
**Histopathology of renal cell carcinoma metastasis in the thyroid: capsulated intrathyroidal nodule (A) composed of nests and cords of large clear cells (B) with abundant optically empty cytoplasm, sharply outlined boundaries and moderately atypical nuclei (C).** The clear cells are CD10-immunoreactive (D). (H&E, ×10, ×100 and ×400; avidin-biotin-peroxidase, ×400).

In addition, as shown in figure 1, nodule with a golden yellow cut surface, measuring 1 cm in its larger dimension, was observed in the left lobe. Microscopically, the nodule was surrounded by a complete fibrous capsule and showed a proliferation of large cells with abundant optically clear cytoplasm and sharply outlined boundaries, arranged in nests and cords. The nuclei showed mild to moderate atypia and single or multiple nucleoli were visible at ×400 magnification. The neoplastic cells were strongly immunoreactive for CD10, which is commonly expressed in renal cell carcinomas. By contrast, TTF-1 was completely negative, and this ruled out a primary tumour of the thyroid. Based on these findings and on the similarity of this proliferation with the renal cell neoplasm diagnosed in the kidney two years before, a diagnosis of a intrathyroid metastasis of renal cell carcinoma, grade II, was made.

Her postoperative course was uneventful.

Postoperative bone scintigraphy and computer tomography also revealed the absence of any other metastases. She has been doing well without any evidence of recurrence for 5 months after thyroidectomy.

## Discussion

The prevalence of intrathyroid metastases of nonthyroid origin ranges from 1.9% to 25% [15-24]. Mortensen et al. account 4% of patients with metastatic neoplasm with secondary tumors to the thyroid gland [25]. Silverberg meticulously examined the gland and found an incidence of 25% in patients dying from disseminated malignant tumor of other primary [26].

Primary carcinomas of the lung, kidney, breast, stomach are the most common tumors metastasizing to the thyroid [15-24]. Carcinomas of the colon, gynecologic tumors, oral cavity, esophagus, neuroendocrine cancers, and sarcomas have been rarely published only in case reports or small series [15-24]. In the study of Shimaoka et al., metastases to the thyroid gland occurred in 39% of melanoma patients, 21% of breast cancer patients, 12% renal cancer, 10% of lung cancer and 10% of patients with primary head and neck tumors [27]. Chen et al. reported ten patients with thyroid metastases and 50% of these patients had metastases from renal cell carcinoma [28]. Thus, renal cell carcinoma is by far the most common source of clinically relevant metastases to the thyroid gland [28].

However, considering that the reported metastases of autopsy cases included nonclinically metastasis (i.e. occult cancer or widespread cancer at the time of death), a better estimate of the incidence of clinically apparent metastases to the thyroid has been shown in clinical studies. The incidence of clinically significant metastases appears to be lower than the incidence found in autopsy. According to Shimaoka, the thyroid metastases were rarely clinically apparent in only 5% to 10% of the patients [27]. Wychulis et al. described that only 10 of 20262 patients, who had undergone thyroidectomy at the Mayo Clinic, had symptomatic metastatic involvement of the thyroid gland [29].

In clinical practice, in a patient with a diffuse and bilateral multinodular goiter, a correct diagnosis is difficult, since there are no specific findings of metastatic thyroid tumor on ultrasonography or computer tomography scan investigations. Elliott and Frantz found 44 reported cases of metastatic carcinoma that had been misidentified as primary thyroid cancer [14].

Therefore, a correct diagnosis of metastatic thyroid tumors requires a careful consideration of patients with a history of cancer. This information immediately stratifies a patient into a high risk category.

Moreover, the presentation of a thyroid nodule years after the treatment of a primary cancer often poses a diagnostic dilemma. Often there is a latency period lasting years between the diagnosis of the primary cancer and the appearance of the thyroid mass [30]. Latent intervals of up to 20 years have also been reported [30]. This finding is especially true for renal primary tumors [30]. In these cases with a long interval between the detection of the primary tumor and the development of the thyroid metastasis, the difficulty in making a correct diagnosis could increase as well.

On the other hand, there have been several reports on metastasis to the thyroid, which appeared prior to the primary tumor being detected [31].

Fine needle aspiration cytology (FNA) can allow for the preoperative diagnosis of a secondary tumor, thus changing the preoperative work-up of such patient [32-34]. Once the diagnosis of metastatic disease has been confirmed on FNA, the patient should undergo a metastatic work-up to rule out other distant metastases [32-34].

Finally, if technically feasible, thyroidectomy can be effective for local control [35]. Surgical resection is regarded as the best treatment for a metastatic thyroid tumor, especially if the primary carcinoma appears to be controlled and there is no evidence of metastasis elsewhere [36]. Moreover, considering the size and rapid growth of the thyroid tumor, even if the patient had already had other metastatic lesions, thyroidectomy would still be required in order to relieve tracheal compression. This is especially true for tumors that present years after the treatment of the primary cancer.

Survival time after diagnosis of the thyroid metastases is determined by the biology of the primary disease [32-36]. Our patient has no evidence of recurrence 5 months after thyroid surgery. Authors have demonstrated that for isolated thyroid metastases, thyroidectomy has prolonged survival. Chen et al. reported that 60% of the patients with solitary thyroid metastasis were still alive after a thyroidectomy during a median follow-up period of 5.2 years [28].

After surgical management, the administration of systemic therapy is recommended [35].

## Authors' contributions

GD: acquisition of data. GD, FR, SU: study conception and design. MG, GD, MLT: analysis and interpretation of data, drafting of manuscript. GD, VB, AL: Critical revision and supervision. The manuscript has been seen and approved by all authors. 

## Competing interests

The authors declare that they have no competing interests.

## Consent

A written consent of the patient was obtained for publication of this report.
